# Successful Use of Extracorporeal Membrane Oxygenation for the Treatment of Cardiogenic Shock due to Scorpion Envenomation

**DOI:** 10.1155/2017/8073989

**Published:** 2017-04-27

**Authors:** Amine Tarmiz, Imene Mgarrech, Chokri Kortas, Sofiane Jerbi

**Affiliations:** Department of Cardiovascular and Thoracic Surgery, Sahloul University Hospital, Sousse, Tunisia

## Abstract

*Introduction*. The occurrence of a cardiogenic shock is a rare presentation after scorpion envenomation. The treatment includes classically the use of inotropes and specific vasodilators.* Case Presentation*. We report a case of an 11-year-old boy presenting with cardiogenic shock and pulmonary edema after a scorpion sting. Despite adequate management at the emergency department and intensive care unit, the patient's hemodynamic status worsened rapidly, justifying his transfer to our department for ventricular mechanical assistance by venoarterial extracorporeal membrane oxygenation. The following outcomes were favorable and the boy was discharged home on day 29 without aftereffects.* Conclusion*. This is the first report of successful use of extracorporeal membrane oxygenation for the treatment of cardiogenic shock after scorpion envenomation.

## 1. Introduction

Scorpion stings occur in many areas of the world, mainly rural regions of countries with a hot and dry climate, affecting predominantly young victims [1].

The majority of these incidents are not serious, with local pain being the only clinical manifestation. However, severe complications such as acute pulmonary edema and cardiogenic shock can occur, mainly in children [1–3].

We herein report a unique case with successful use of extracorporeal membrane oxygenation (ECMO) for the treatment of cardiogenic shock due to scorpion envenomation in a child.

## 2. Case Presentation

An 11-year-old male was initially admitted to an emergency department after being stung in the left hand by a scorpion while playing in front of his family house in an endemic area of our country.

A few minutes after the sting, he experienced severe local pain, erythema, and numbness of his left arm, as well as several episodes of profuse sweating and vomiting.

Initial clinical examination showed blood pressure of 190 mmHg/110 mmHg and a heart rate of 140 bpm. Electrocardiogram revealed sinus rhythm with no ST-T modifications. He received analgesic and primary medical care.

**Figure 1:**
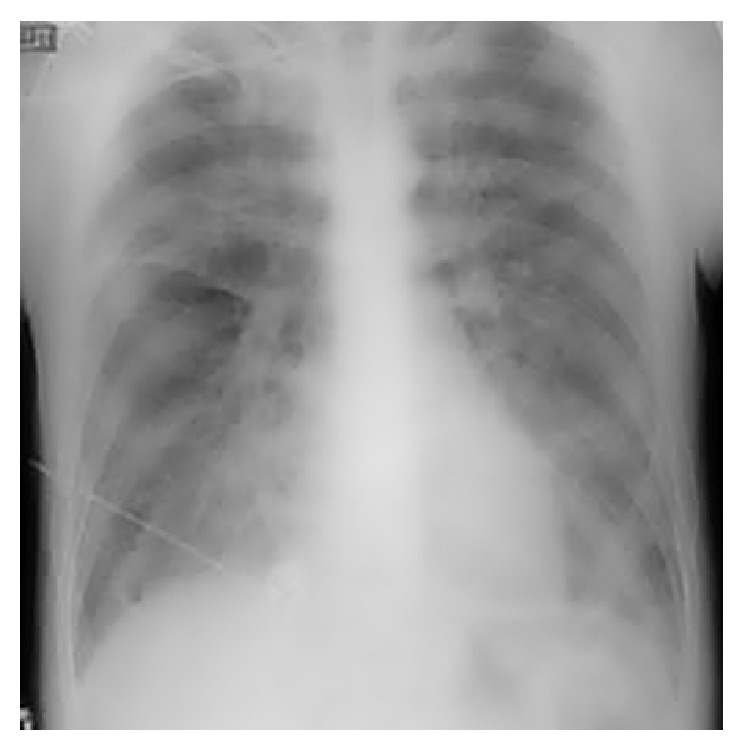
Chest radiograph showing pulmonary edema with normal cardiac area.

Upon admission, moderate respiratory distress was noticed with oxygen saturation of 90% in room air associated with bilateral rales compatible with acute pulmonary edema. Chest X-ray showed bilateral fluffy shadows compatible with pulmonary edema with normal cardiac area (Figure 1).

The child was thus referred to the pediatric intensive care unit of the same hospital. During the first hour after his transfer, he presented hypotension (68 × 38 mmHg), poor peripheral perfusion, and worsening of respiratory distress. The diagnosis of cardiogenic shock with pulmonary edema was made and dobutamine was started at the rate of 10 *γ*/kg/min.

Transthoracic echocardiography demonstrated severe global hypokinesia of both right and left ventricles with elevated filling pressures and left ventricle ejection fraction of 25%. Initial laboratory tests showed metabolic acidosis with lactate elevation and troponin I elevation (Table 1).

Because of this severe presentation, scorpion antivenom was administered intravenously about 4 hours after the incident.

After this intervention, the hemodynamic status of the patient worsened despite raising dobutamine doses (20 *γ*/kg/min) and adrenaline (0,3 *γ*/kg/min) adjunction.

**Figure 2:**
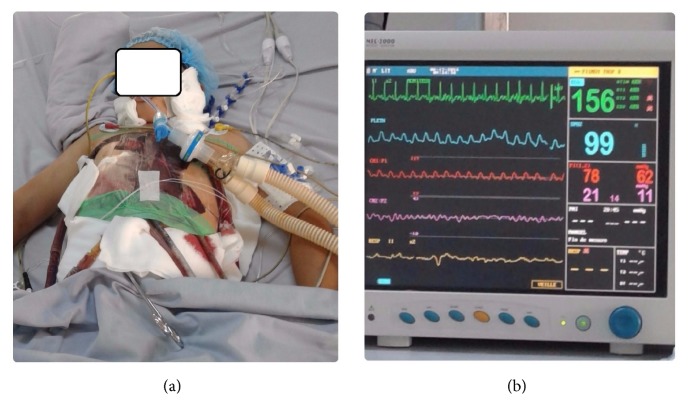
(a) Postoperative view of the central venoarterial ECMO in the patient. (b) Hemodynamic status monitoring under ECMO with pulsatile arterial pressure.

The patient was immediately transferred to our department for ventricular mechanical assistance by venoarterial ECMO (Figure 2).

Intrathoracic cannulation was performed to avoid peripheral ECMO complications, especially left ventricle (LV) distention. For this purpose, active LV drainage was obtained by placing a cannula in the left atrium.

The venous cannula was placed in the right atrium and the outflow from the ECMO was placed in the ascending aorta.

After this intervention, the hemodynamic status of the patient improved considerably and both dobutamine and adrenaline were discontinued.

ECMO assistance was maintained for 5 days and weaning off ECMO was obtained with dobutamine 5 *γ*/kg/min. At that time, echocardiography showed considerable improvement of the cardiac function with LVEF of 45%.

On day 12, the patient developed postmedian sternotomy mediastinitis that was treated surgically.

He was discharged home on day 29 without aftereffects.

## 3. Discussion

More than one million cases of scorpion envenomation are yearly reported worldwide [1]. Although most cases are minor, with localized pain and minimal systemic involvement, severe envenomation is a major public health problem in certain parts of the world: Central and South America, North Africa, the Middle East, and South Asia [2]. Most scorpions that cause serious medical problems belong to the Buthidae family, which especially includes scorpions from* Androctonus* and* Buthus* in North Africa. With regard to witnesses' description in our case, culpability of* Androctonus australis* is highly suspected. This scorpion is responsible for a high rate of severe envenomation with cardiorespiratory manifestations [2].

The symptomatology of scorpion envenomation is polymorphous. Bouaziz et al. [3] stratified patients into three grades of severity at baseline, according to the absence or presence of systemic manifestations [3]. Grade I included patients who had only localized manifestations of scorpion sting; grade II included patients who also had systemic manifestations; and grade III included patients with cardiorespiratory manifestations, mainly cardiogenic shock and pulmonary edema or severe neurological manifestation (coma and/or convulsion). A recent consensus report defined four classes of scorpion envenomation: local, minor, major, and lethal [4]. The last class is defined by multiorgan failure with necessity of mechanical ventilation, inotropes, and benzodiazepine infusion. In Bouaziz et al.'s study [3], 61.5% of patients had a pulmonary edema, while 20.5% of patients had a cardiogenic shock.

Cardiorespiratory manifestations, mainly cardiogenic shock and pulmonary edema, are the leading causes of death after scorpion envenomation. Almost 40 000 stung patients are recorded in Tunisia each year; 1000 of these have systemic manifestations requiring admission to a hospital, of whom about 10 patients die [5]. Cardiac consequences occur as a result of sympathetic excitation and the release of catecholamine in the plasma and also in the myocardium. In the initial period, there is an increase in blood pressure and cardiac output, followed by diminished left ventricular function and hypotension. Possible mechanisms of myocardial ischemia include imbalance in blood pressure and coronary vasospasm caused by the combination of sympathetic excitation and release of catecholamines induced by scorpion venom and the direct effect of the toxin on the myocardium [2]. Furthermore, in cases of myocardial infarction after a scorpion sting, vasoactive, inflammatory, and thrombogenic substances, such as histamine, serotonin, bradykinin, leukotrienes, and thromboxane, are released. These substances act on the coronary vasculature and may induce coronary artery vasospasm and platelet aggregation and facilitate thrombus formation [6].

Regarding treatment, in severe envenomation, the standard intensive care treatment for acute pulmonary edema and cardiogenic shock appears to be appropriate and often includes the use of inotropes (e.g., dobutamine) and specific vasodilators (e.g., prazosin) [7].

Administration of scorpion antivenom, which only neutralizes circulating toxin, is recommended in moderate and severe cases. Although its efficacy and benefit are controversial, this antivenom is more efficient when given early. Our patient received the antivenom about four hours after the incident, a period considered to represent a good time window for treatment [8]. In some refractory and “lethal” cases, supportive care and mechanical ventilation may be necessary to support the cardiorespiratory system. In these specific cases and to avoid multiorgan failure, seizures, and end-organ damage caused by hypotension, we believe that ECMO could be an interesting way to manage these patients, as a bridge to recovery. Indeed, it allows supporting the cardiorespiratory system to get over the catecholaminergic storm and the circulating toxins. In our case, once ECMO was started, the patient's hemodynamic status improved rapidly and it took us 5 days to wean the child off the ECMO.

To the best of our knowledge, the use of ECMO for the treatment of cardiogenic shock after scorpion envenomation has not been reported previously. Nonetheless, we strongly believe that the use of ECMO in severe scorpion envenomation with cardiogenic shock should be evoked at the convenient moment to prevent multiorgan failure.

## Figures and Tables

**Table 1:** Laboratory findings on admission and during follow-up.

Variables	Admission	24 hours	Day 5
Glucose (mg/dL)	458	174	98
Lactate (mmol/L)	12,8	9,6	1,2
Arterial pH	7,08	7,37	7,42
Bicarbonate (mEq/L)	11	20	25
BE	−14	−6	1
pCO_2_ (mmHg)	24	37	39
pO_2_ (mmHg)	67	241	201
CK-MB (ng/mL)	60,8	300	2,65
Troponin I (ng/mL)	13	68	2,4
